# 1-Benzyl-*N*-methyl-1*H*-pyrrole-2-carboxamide

**DOI:** 10.1107/S160053681002787X

**Published:** 2010-07-17

**Authors:** Xiang Chao Zeng, Kai Ping Li, Fang Hu, Le Zheng

**Affiliations:** a Department of Chemistry, Jinan University, Guangzhou, Guangdong 510632, People’s Republic of China

## Abstract

The asymmetric unit of the title compound, C_13_H_14_N_2_O, contains two independent mol­ecules, which differ in the twist of the phenyl ring: the N_pyrrole_—C(H_2_)—C—C torsion angles are −73.0 (3) and 17.1 (3)°. In the crystal structure, mol­ecules are linked through N—H⋯O hydrogen bonds into chains extending along the *a* axis.

## Related literature

For the bioactivity of pyrrole derivatives, see: Fabio *et al.* (2007 ▶); Banwell *et al.* (2006 ▶). For related structures, see: Zeng *et al.* (2007 ▶); Li *et al.* (2009 ▶).
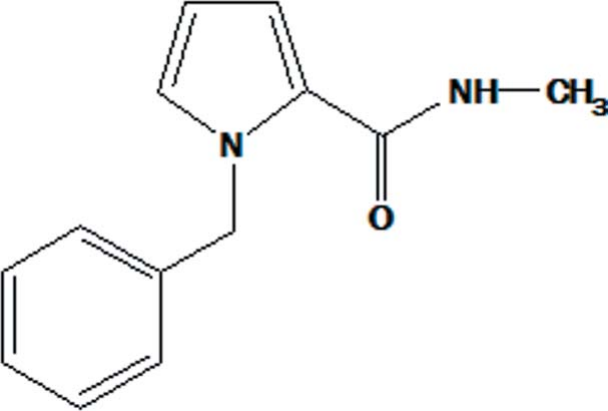


## Experimental

### 

#### Crystal data

C_13_H_14_N_2_O
*M* *_r_* = 214.26
Monoclinic, 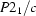

*a* = 9.8285 (18) Å
*b* = 23.588 (4) Å
*c* = 9.9230 (17) Å
β = 90.107 (3)°
*V* = 2300.5 (7) Å^3^
*Z* = 8
Mo *K*α radiation
μ = 0.08 mm^−1^
*T* = 110 K
0.45 × 0.43 × 0.41 mm

#### Data collection

Bruker SMART 1K CCD area-detector diffractometer
Absorption correction: multi-scan (*SADABS*; Sheldrick, 1996 ▶) *T*_min_ = 0.965, *T*_max_ = 0.968
10728 measured reflections
4879 independent reflections
4388 reflections with *I* > 2σ(*I*)
*R*_int_ = 0.033

#### Refinement

*R*[*F* ^2^ > 2σ(*F* ^2^)] = 0.055
*wR*(*F* ^2^) = 0.157
*S* = 1.03
4879 reflections
300 parameters
H atoms treated by a mixture of independent and constrained refinement
Δρ_max_ = 0.24 e Å^−3^
Δρ_min_ = −0.32 e Å^−3^

### 

Data collection: *SMART* (Bruker, 1999 ▶); cell refinement: *SAINT-Plus* (Bruker, 1999 ▶); data reduction: *SAINT-Plus*; program(s) used to solve structure: *SHELXS97* (Sheldrick, 2008 ▶); program(s) used to refine structure: *SHELXL97* (Sheldrick, 2008 ▶); molecular graphics: *SHELXTL* (Sheldrick, 2008 ▶); software used to prepare material for publication: *SHELXTL*.

## Supplementary Material

Crystal structure: contains datablocks I, global. DOI: 10.1107/S160053681002787X/cv2741sup1.cif

Structure factors: contains datablocks I. DOI: 10.1107/S160053681002787X/cv2741Isup2.hkl

Additional supplementary materials:  crystallographic information; 3D view; checkCIF report

## Figures and Tables

**Table 1:** Hydrogen-bond geometry (Å, °)

*D*—H⋯*A*	*D*—H	H⋯*A*	*D*⋯*A*	*D*—H⋯*A*
N4—H4⋯O1^i^	0.87 (3)	2.04 (3)	2.869 (2)	161 (3)
N2—H2*A*⋯O3^ii^	0.86 (4)	2.10 (4)	2.902 (2)	154 (3)

Symmetry codes: (i) 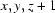; (ii) 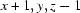.

**Fig. 1.**
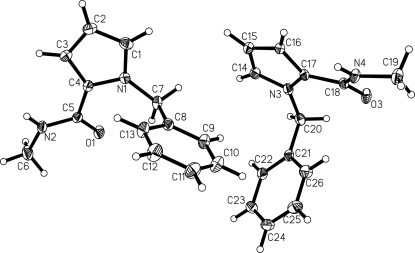
Two independent molecules of the title compound, with the atom-numbering scheme. Displacement ellipsoids are drawn at the 30% probability level.

**Fig. 2.**
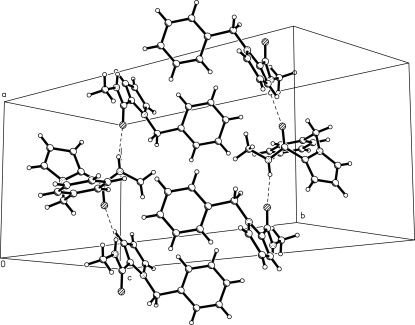
Crystal packing of (I) showing the chains formed by hydrogen bonds (dashed lines).

## supplementary crystallographic information

### Comment

Many pyrrole derivatives show important bioactivities, such as metabotropic receptor antagonists (Fabio *et al.*, 2007) and antitumor activity (Banwell *et al.*, 2006). This is the reason they have attracted our interest. This study is related to our previous structural investigations of methyl 2-(4,5-dibromo-1*H*-pyrrole-2-carboxamido)propionate (Zeng *et al.*, 2007) and 3-(1-ethyl-1*H*-pyrrole-2-carboxamido) propionic acid monohydrate (Li *et al.*, 2009).

In the molecule of the title compound(Fig.1), bond lengths and angles are unexceptional. In the crystal structure, molecules are linked through N—H···O hydrogen bonds, forming chains extending to the *a* axis (Fig. 2).

### Experimental

A suspension of potassium carbonate (4.21 g, 30 mmol), chloromethylbenzene (1.7 ml, 15 mmol), Pyrrole-2-carboxylic acid methylamide (1.24 g, 10 mmol) and Tetrabutylammoniumbromide (0.1 g) in acetonitrile (25 ml) magnetically stirred at 353 K for 18 h. After filtration, the filtrate was evaporated *in vacuo*, and the crude compound (I) was obtained. The impure product was dissolved in EtOH, colourless crystals suitable for X-ray analysis, m.p. 365 K, 92.1%, were obtained over a period of one week by slow evaporation at room temperature of the solution.

### Refinement

The H atoms bonded to N2 and N4 were found on a difference Fourier map and refined isotropically with N—H = 0.86 (4)Å and 0.87 (3)Å respectively. Remaining H atoms were positioned geometrically [C—H = 0.99Å for CH_2_, 0.98Å for CH_3_ and 0.95Å for CH(aromatic)] and refined using a riding model, with *U*_iso_ = 1.2*U*_eq_ (1.5*U*_eq_ for the methyl group) of the parent atom.

### Crystal data

**Table:**

C_13_H_14_N_2_O	*D*_x_ = 1.237 Mg m^−^^3^
*M**_r_* = 214.26	Melting point: 365 K
Monoclinic, *P*2_1_/*c*	Mo *K*α radiation, λ = 0.71073 Å
*a* = 9.8285 (18) Å	Cell parameters from 7113 reflections
*b* = 23.588 (4) Å	θ = 2.2–27.0°
*c* = 9.9230 (17) Å	µ = 0.08 mm^−^^1^
β = 90.107 (3)°	*T* = 110 K
*V* = 2300.5 (7) Å^3^	Prism, colourless
*Z* = 8	0.45 × 0.43 × 0.41 mm
*F*(000) = 912	

### Data collection

**Table:**

Bruker SMART 1K CCD area-detector diffractometer	4879 independent reflections
Radiation source: fine-focus sealed tube	4388 reflections with *I* > 2σ(*I*)
graphite	*R*_int_ = 0.033
φ and ω scans	θ_max_ = 27.0°, θ_min_ = 1.7°
Absorption correction: multi-scan (*SADABS*; Sheldrick, 1996)	*h* = −12→12
*T*_min_ = 0.965, *T*_max_ = 0.968	*k* = −30→18
10728 measured reflections	*l* = −8→12

### Refinement

**Table:**

Refinement on *F*^2^	Primary atom site location: structure-invariant direct methods
Least-squares matrix: full	Secondary atom site location: difference Fourier map
*R*[*F*^2^ > 2σ(*F*^2^)] = 0.055	Hydrogen site location: inferred from neighbouring sites
*wR*(*F*^2^) = 0.157	H atoms treated by a mixture of independent and constrained refinement
*S* = 1.02	*w* = 1/[σ^2^(*F*_o_^2^) + (0.1044*P*)^2^ + 0.2726*P*] where *P* = (*F*_o_^2^ + 2*F*_c_^2^)/3
4879 reflections	(Δ/σ)_max_ = 0.001
300 parameters	Δρ_max_ = 0.24 e Å^−^^3^
0 restraints	Δρ_min_ = −0.32 e Å^−^^3^

### Special details

**Table:**

Geometry. All e.s.d.'s (except the e.s.d. in the dihedral angle between two l.s. planes) are estimated using the full covariance matrix. The cell e.s.d.'s are taken into account individually in the estimation of e.s.d.'s in distances, angles and torsion angles; correlations between e.s.d.'s in cell parameters are only used when they are defined by crystal symmetry. An approximate (isotropic) treatment of cell e.s.d.'s is used for estimating e.s.d.'s involving l.s. planes.
Refinement. Refinement of *F*^2^ against ALL reflections. The weighted *R*-factor *wR* and goodness of fit *S* are based on *F*^2^, conventional *R*-factors *R* are based on *F*, with *F* set to zero for negative *F*^2^. The threshold expression of *F*^2^ > σ(*F*^2^) is used only for calculating *R*-factors(gt) *etc*. and is not relevant to the choice of reflections for refinement. *R*-factors based on *F*^2^ are statistically about twice as large as those based on *F*, and *R*- factors based on ALL data will be even larger.

### Fractional atomic coordinates and isotropic or equivalent isotropic displacement parameters (Å^2^)

**Table:**

	*x*	*y*	*z*	*U*_iso_*/*U*_eq_	
O3	0.26038 (14)	0.34460 (7)	1.12858 (15)	0.0345 (4)	
N4	0.46989 (19)	0.36072 (8)	1.21438 (18)	0.0298 (4)	
O1	0.75946 (15)	0.35421 (7)	0.24801 (16)	0.0351 (4)	
C18	0.38669 (19)	0.34269 (8)	1.11684 (19)	0.0248 (4)	
N3	0.38660 (18)	0.31280 (7)	0.87447 (16)	0.0278 (4)	
C5	0.8847 (2)	0.34954 (8)	0.2623 (2)	0.0261 (4)	
C4	0.94580 (19)	0.32331 (7)	0.3830 (2)	0.0251 (4)	
N1	0.87834 (18)	0.31770 (7)	0.50486 (17)	0.0284 (4)	
C3	1.0677 (2)	0.29404 (8)	0.3945 (2)	0.0299 (4)	
H3	1.1347	0.2903	0.3262	0.036*	
C21	0.2777 (2)	0.39956 (8)	0.7816 (2)	0.0276 (4)	
C17	0.4525 (2)	0.31943 (8)	0.9961 (2)	0.0254 (4)	
N2	0.97245 (19)	0.36613 (8)	0.16790 (19)	0.0325 (4)	
C7	0.7549 (2)	0.34689 (9)	0.5492 (2)	0.0318 (4)	
H7A	0.7010	0.3207	0.6058	0.038*	
H7B	0.6994	0.3564	0.4690	0.038*	
C16	0.5796 (2)	0.29413 (8)	0.9852 (2)	0.0305 (4)	
H16	0.6469	0.2920	1.0539	0.037*	
C25	0.3206 (3)	0.49865 (10)	0.8254 (3)	0.0427 (5)	
H25	0.3390	0.5284	0.8873	0.051*	
C15	0.5903 (3)	0.27231 (9)	0.8536 (2)	0.0378 (5)	
H15	0.6659	0.2525	0.8170	0.045*	
C8	0.7817 (2)	0.40061 (8)	0.6283 (2)	0.0289 (4)	
C20	0.2580 (2)	0.33927 (9)	0.8305 (2)	0.0305 (4)	
H20A	0.1931	0.3393	0.9067	0.037*	
H20B	0.2178	0.3163	0.7569	0.037*	
C19	0.4191 (2)	0.37479 (11)	1.3473 (2)	0.0387 (5)	
H19A	0.3238	0.3867	1.3404	0.058*	
H19B	0.4735	0.4057	1.3855	0.058*	
H19C	0.4256	0.3414	1.4058	0.058*	
C11	0.8202 (3)	0.50016 (11)	0.7752 (3)	0.0442 (5)	
H11	0.8334	0.5341	0.8251	0.053*	
C1	0.9583 (3)	0.28673 (8)	0.5899 (2)	0.0355 (5)	
H1	0.9366	0.2775	0.6805	0.043*	
C14	0.4714 (3)	0.28490 (8)	0.7884 (2)	0.0345 (5)	
H14	0.4511	0.2757	0.6974	0.041*	
C22	0.2682 (2)	0.41183 (9)	0.6454 (2)	0.0338 (5)	
H22	0.2501	0.3822	0.5830	0.041*	
C26	0.3042 (2)	0.44347 (9)	0.8715 (2)	0.0371 (5)	
H26	0.3111	0.4357	0.9652	0.044*	
C6	0.9276 (3)	0.38588 (13)	0.0361 (3)	0.0497 (6)	
H6A	0.8989	0.3534	−0.0186	0.075*	
H6B	1.0027	0.4055	−0.0090	0.075*	
H6C	0.8510	0.4120	0.0470	0.075*	
C9	0.6769 (2)	0.42377 (10)	0.7042 (2)	0.0379 (5)	
H9	0.5909	0.4054	0.7066	0.045*	
C2	1.0749 (2)	0.27108 (8)	0.5242 (2)	0.0347 (5)	
H2	1.1469	0.2488	0.5601	0.042*	
C13	0.9061 (2)	0.42821 (10)	0.6273 (2)	0.0378 (5)	
H13	0.9790	0.4132	0.5756	0.045*	
C23	0.2847 (3)	0.46671 (10)	0.5993 (2)	0.0423 (5)	
H23	0.2785	0.4746	0.5056	0.051*	
C10	0.6962 (3)	0.47337 (11)	0.7765 (3)	0.0447 (6)	
H10	0.6233	0.4889	0.8272	0.054*	
C12	0.9253 (3)	0.47759 (11)	0.7011 (3)	0.0469 (6)	
H12	1.0114	0.4959	0.7004	0.056*	
C24	0.3101 (3)	0.51017 (10)	0.6894 (3)	0.0426 (5)	
H24	0.3203	0.5479	0.6577	0.051*	
H2A	1.058 (4)	0.3631 (12)	0.183 (3)	0.051 (8)*	
H4	0.557 (3)	0.3597 (11)	1.204 (3)	0.044 (7)*	

### Atomic displacement parameters (Å^2^)

**Table:**

	*U*^11^	*U*^22^	*U*^33^	*U*^12^	*U*^13^	*U*^23^
O3	0.0196 (6)	0.0549 (9)	0.0290 (7)	−0.0027 (6)	0.0040 (6)	−0.0056 (7)
N4	0.0200 (8)	0.0428 (9)	0.0267 (8)	0.0019 (7)	0.0021 (7)	−0.0066 (7)
O1	0.0200 (7)	0.0510 (9)	0.0342 (7)	0.0021 (6)	−0.0005 (6)	0.0048 (7)
C18	0.0228 (8)	0.0286 (8)	0.0231 (8)	−0.0006 (7)	0.0033 (8)	0.0014 (7)
N3	0.0334 (9)	0.0271 (7)	0.0230 (7)	−0.0028 (6)	0.0040 (7)	−0.0011 (6)
C5	0.0221 (9)	0.0259 (8)	0.0301 (9)	0.0001 (7)	0.0010 (8)	−0.0020 (8)
C4	0.0235 (9)	0.0258 (8)	0.0261 (9)	−0.0012 (7)	0.0017 (8)	−0.0031 (7)
N1	0.0292 (8)	0.0297 (8)	0.0263 (8)	0.0001 (7)	0.0027 (7)	0.0007 (7)
C3	0.0288 (9)	0.0297 (9)	0.0311 (10)	0.0050 (7)	−0.0019 (9)	−0.0038 (8)
C21	0.0216 (8)	0.0337 (10)	0.0276 (9)	0.0015 (7)	0.0028 (8)	−0.0002 (8)
C17	0.0246 (9)	0.0269 (8)	0.0249 (9)	−0.0021 (7)	0.0063 (8)	0.0023 (7)
N2	0.0220 (8)	0.0439 (10)	0.0315 (9)	0.0032 (7)	0.0047 (7)	0.0066 (8)
C7	0.0251 (10)	0.0390 (11)	0.0313 (9)	−0.0027 (8)	0.0067 (9)	−0.0027 (8)
C16	0.0296 (10)	0.0313 (9)	0.0307 (10)	0.0058 (8)	0.0091 (9)	0.0047 (8)
C25	0.0439 (12)	0.0329 (10)	0.0512 (13)	0.0004 (9)	−0.0023 (12)	−0.0046 (10)
C15	0.0473 (13)	0.0321 (9)	0.0339 (11)	0.0097 (9)	0.0157 (10)	0.0006 (9)
C8	0.0275 (9)	0.0339 (9)	0.0251 (8)	0.0022 (8)	0.0007 (8)	0.0024 (8)
C20	0.0309 (10)	0.0345 (10)	0.0260 (8)	−0.0067 (8)	0.0001 (8)	−0.0001 (8)
C19	0.0307 (11)	0.0574 (13)	0.0280 (10)	0.0063 (10)	0.0003 (9)	−0.0112 (9)
C11	0.0467 (13)	0.0429 (12)	0.0431 (12)	0.0042 (10)	−0.0025 (11)	−0.0125 (10)
C1	0.0455 (13)	0.0323 (10)	0.0287 (10)	0.0005 (9)	−0.0043 (10)	0.0017 (8)
C14	0.0493 (13)	0.0276 (10)	0.0265 (9)	0.0036 (9)	0.0080 (10)	−0.0031 (8)
C22	0.0370 (11)	0.0386 (11)	0.0260 (9)	0.0064 (9)	0.0037 (8)	−0.0001 (9)
C26	0.0419 (12)	0.0367 (10)	0.0325 (10)	−0.0014 (9)	−0.0046 (10)	−0.0030 (9)
C6	0.0376 (12)	0.0753 (17)	0.0362 (11)	0.0101 (12)	0.0077 (11)	0.0211 (12)
C9	0.0288 (10)	0.0461 (12)	0.0387 (11)	0.0016 (9)	0.0055 (10)	−0.0029 (10)
C2	0.0395 (11)	0.0315 (10)	0.0329 (10)	0.0082 (9)	−0.0063 (9)	−0.0026 (8)
C13	0.0272 (10)	0.0441 (11)	0.0421 (11)	−0.0009 (9)	0.0057 (10)	−0.0089 (10)
C23	0.0497 (14)	0.0452 (12)	0.0320 (10)	0.0104 (10)	0.0063 (11)	0.0113 (10)
C10	0.0369 (12)	0.0518 (14)	0.0454 (13)	0.0097 (10)	0.0050 (11)	−0.0117 (11)
C12	0.0347 (12)	0.0497 (13)	0.0563 (15)	−0.0080 (10)	0.0001 (12)	−0.0131 (12)
C24	0.0402 (12)	0.0340 (11)	0.0536 (13)	0.0057 (9)	0.0065 (11)	0.0103 (10)

### Geometric parameters (Å, °)

**Table:**

O3—C18	1.248 (2)	C15—C14	1.367 (4)
N4—C18	1.335 (3)	C15—H15	0.9500
N4—C19	1.450 (3)	C8—C13	1.386 (3)
N4—H4	0.87 (3)	C8—C9	1.390 (3)
O1—C5	1.243 (2)	C20—H20A	0.9900
C18—C17	1.469 (3)	C20—H20B	0.9900
N3—C14	1.364 (3)	C19—H19A	0.9800
N3—C17	1.378 (3)	C19—H19B	0.9800
N3—C20	1.475 (3)	C19—H19C	0.9800
C5—N2	1.334 (3)	C11—C10	1.373 (4)
C5—C4	1.475 (3)	C11—C12	1.376 (4)
C4—N1	1.386 (3)	C11—H11	0.9500
C4—C3	1.388 (3)	C1—C2	1.369 (3)
N1—C1	1.364 (3)	C1—H1	0.9500
N1—C7	1.463 (3)	C14—H14	0.9500
C3—C2	1.398 (3)	C22—C23	1.383 (3)
C3—H3	0.9500	C22—H22	0.9500
C21—C22	1.385 (3)	C26—H26	0.9500
C21—C26	1.391 (3)	C6—H6A	0.9800
C21—C20	1.515 (3)	C6—H6B	0.9800
C17—C16	1.389 (3)	C6—H6C	0.9800
N2—C6	1.456 (3)	C9—C10	1.385 (3)
N2—H2A	0.86 (4)	C9—H9	0.9500
C7—C8	1.513 (3)	C2—H2	0.9500
C7—H7A	0.9900	C13—C12	1.388 (3)
C7—H7B	0.9900	C13—H13	0.9500
C16—C15	1.408 (3)	C23—C24	1.383 (4)
C16—H16	0.9500	C23—H23	0.9500
C25—C24	1.381 (4)	C10—H10	0.9500
C25—C26	1.389 (3)	C12—H12	0.9500
C25—H25	0.9500	C24—H24	0.9500
			
C18—N4—C19	121.39 (18)	N3—C20—H20B	109.1
C18—N4—H4	120.7 (19)	C21—C20—H20B	109.1
C19—N4—H4	117.3 (19)	H20A—C20—H20B	107.8
O3—C18—N4	121.92 (18)	N4—C19—H19A	109.5
O3—C18—C17	121.96 (18)	N4—C19—H19B	109.5
N4—C18—C17	116.10 (17)	H19A—C19—H19B	109.5
C14—N3—C17	108.44 (18)	N4—C19—H19C	109.5
C14—N3—C20	122.93 (18)	H19A—C19—H19C	109.5
C17—N3—C20	127.76 (16)	H19B—C19—H19C	109.5
O1—C5—N2	122.4 (2)	C10—C11—C12	119.6 (2)
O1—C5—C4	122.11 (19)	C10—C11—H11	120.2
N2—C5—C4	115.51 (18)	C12—C11—H11	120.2
N1—C4—C3	107.17 (18)	N1—C1—C2	109.37 (19)
N1—C4—C5	123.61 (17)	N1—C1—H1	125.3
C3—C4—C5	128.74 (19)	C2—C1—H1	125.3
C1—N1—C4	108.33 (18)	N3—C14—C15	109.37 (19)
C1—N1—C7	122.91 (18)	N3—C14—H14	125.3
C4—N1—C7	127.97 (17)	C15—C14—H14	125.3
C4—C3—C2	108.07 (19)	C23—C22—C21	120.7 (2)
C4—C3—H3	126.0	C23—C22—H22	119.6
C2—C3—H3	126.0	C21—C22—H22	119.6
C22—C21—C26	118.8 (2)	C25—C26—C21	120.5 (2)
C22—C21—C20	120.01 (18)	C25—C26—H26	119.7
C26—C21—C20	121.14 (18)	C21—C26—H26	119.7
N3—C17—C16	107.72 (18)	N2—C6—H6A	109.5
N3—C17—C18	123.36 (17)	N2—C6—H6B	109.5
C16—C17—C18	128.44 (19)	H6A—C6—H6B	109.5
C5—N2—C6	122.00 (19)	N2—C6—H6C	109.5
C5—N2—H2A	119 (2)	H6A—C6—H6C	109.5
C6—N2—H2A	118 (2)	H6B—C6—H6C	109.5
N1—C7—C8	113.97 (17)	C10—C9—C8	120.8 (2)
N1—C7—H7A	108.8	C10—C9—H9	119.6
C8—C7—H7A	108.8	C8—C9—H9	119.6
N1—C7—H7B	108.8	C1—C2—C3	107.04 (19)
C8—C7—H7B	108.8	C1—C2—H2	126.5
H7A—C7—H7B	107.7	C3—C2—H2	126.5
C17—C16—C15	107.3 (2)	C8—C13—C12	120.6 (2)
C17—C16—H16	126.3	C8—C13—H13	119.7
C15—C16—H16	126.3	C12—C13—H13	119.7
C24—C25—C26	119.9 (2)	C24—C23—C22	120.1 (2)
C24—C25—H25	120.1	C24—C23—H23	120.0
C26—C25—H25	120.1	C22—C23—H23	120.0
C14—C15—C16	107.11 (19)	C11—C10—C9	120.3 (2)
C14—C15—H15	126.4	C11—C10—H10	119.8
C16—C15—H15	126.4	C9—C10—H10	119.8
C13—C8—C9	118.3 (2)	C11—C12—C13	120.4 (2)
C13—C8—C7	122.84 (19)	C11—C12—H12	119.8
C9—C8—C7	118.81 (19)	C13—C12—H12	119.8
N3—C20—C21	112.46 (16)	C25—C24—C23	119.9 (2)
N3—C20—H20A	109.1	C25—C24—H24	120.0
C21—C20—H20A	109.1	C23—C24—H24	120.0
			
C19—N4—C18—O3	10.3 (3)	N1—C7—C8—C9	−163.65 (18)
C19—N4—C18—C17	−168.55 (18)	C14—N3—C20—C21	−87.5 (2)
O1—C5—C4—N1	−20.5 (3)	C17—N3—C20—C21	80.6 (2)
N2—C5—C4—N1	161.67 (17)	C22—C21—C20—N3	107.6 (2)
O1—C5—C4—C3	150.5 (2)	C26—C21—C20—N3	−73.0 (3)
N2—C5—C4—C3	−27.3 (3)	C4—N1—C1—C2	−1.4 (2)
C3—C4—N1—C1	1.2 (2)	C7—N1—C1—C2	−171.95 (18)
C5—C4—N1—C1	173.90 (17)	C17—N3—C14—C15	1.3 (2)
C3—C4—N1—C7	171.15 (18)	C20—N3—C14—C15	171.34 (17)
C5—C4—N1—C7	−16.2 (3)	C16—C15—C14—N3	−0.9 (2)
N1—C4—C3—C2	−0.6 (2)	C26—C21—C22—C23	0.0 (3)
C5—C4—C3—C2	−172.77 (19)	C20—C21—C22—C23	179.4 (2)
C14—N3—C17—C16	−1.1 (2)	C24—C25—C26—C21	0.2 (4)
C20—N3—C17—C16	−170.53 (17)	C22—C21—C26—C25	0.1 (3)
C14—N3—C17—C18	−173.73 (17)	C20—C21—C26—C25	−179.3 (2)
C20—N3—C17—C18	16.8 (3)	C13—C8—C9—C10	0.3 (3)
O3—C18—C17—N3	18.7 (3)	C7—C8—C9—C10	−179.0 (2)
N4—C18—C17—N3	−162.52 (17)	N1—C1—C2—C3	1.0 (2)
O3—C18—C17—C16	−152.4 (2)	C4—C3—C2—C1	−0.2 (2)
N4—C18—C17—C16	26.4 (3)	C9—C8—C13—C12	0.4 (4)
O1—C5—N2—C6	−6.0 (3)	C7—C8—C13—C12	179.6 (2)
C4—C5—N2—C6	171.8 (2)	C21—C22—C23—C24	−0.4 (4)
C1—N1—C7—C8	75.4 (2)	C12—C11—C10—C9	0.3 (4)
C4—N1—C7—C8	−93.1 (2)	C8—C9—C10—C11	−0.6 (4)
N3—C17—C16—C15	0.5 (2)	C10—C11—C12—C13	0.4 (4)
C18—C17—C16—C15	172.66 (19)	C8—C13—C12—C11	−0.7 (4)
C17—C16—C15—C14	0.3 (2)	C26—C25—C24—C23	−0.7 (4)
N1—C7—C8—C13	17.1 (3)	C22—C23—C24—C25	0.8 (4)

### Hydrogen-bond geometry (Å, °)

**Table:**

*D*—H···*A*	*D*—H	H···*A*	*D*···*A*	*D*—H···*A*
N4—H4···O1^i^	0.87 (3)	2.04 (3)	2.869 (2)	161 (3)
N2—H2A···O3^ii^	0.86 (4)	2.10 (4)	2.902 (2)	154 (3)

Symmetry codes: (i) *x*, *y*, *z*+1; (ii) *x*+1, *y*, *z*−1.
